# Role of autophagy in cell-penetrating peptide transfection model

**DOI:** 10.1038/s41598-017-12747-z

**Published:** 2017-10-03

**Authors:** Moataz Dowaidar, Maxime Gestin, Carmine Pasquale Cerrato, Mohammed Hakim Jafferali, Helerin Margus, Paula Ann Kivistik, Kariem Ezzat, Einar Hallberg, Margus Pooga, Mattias Hällbrink, Ülo Langel

**Affiliations:** 1 Department of Neurochemistry, The Arrhenius Laboratories for Natural Sciences, Stockholm University, Svante Arrhenius väg 16B, SE-10691 Stockholm, Sweden; 2 Department of Developmental Biology, Institute of Molecular and Cell Biology, University of Tartu, 23 Riia Street, 51010 Tartu, Estonia; 3 Laboratory of Molecular Biotechnology, Institute of Technology, University of Tartu, Nooruse, 50411 Tartu, Estonia; 4 Estonian Genome Center, University of Tartu, Tartu, Estonia; 5 Department of Laboratory Medicine, Karolinska Institutet, Huddinge, Sweden

## Abstract

Cell-penetrating peptides (CPPs) uptake mechanism is still in need of more clarification to have a better understanding of their action in the mediation of oligonucleotide transfection. In this study, the effect on early events (1 h treatment) in transfection by PepFect14 (PF14), with or without oligonucleotide cargo on gene expression, in HeLa cells, have been investigated. The RNA expression profile was characterized by RNA sequencing and confirmed by qPCR analysis. The gene regulations were then related to the biological processes by the study of signaling pathways that showed the induction of autophagy-related genes in early transfection. A ligand library interfering with the detected intracellular pathways showed concentration-dependent effects on the transfection efficiency of splice correction oligonucleotide complexed with PepFect14, proving that the autophagy process is induced upon the uptake of complexes. Finally, the autophagy induction and colocalization with autophagosomes have been confirmed by confocal microscopy and transmission electron microscopy. We conclude that autophagy, an inherent cellular response process, is triggered by the cellular uptake of CPP-based transfection system. This finding opens novel possibilities to use autophagy modifiers in future gene therapy.

## Introduction

Gene therapy aims to deliver gene modulating agents into the cells to restore, modify, or silence the function of mutant genes^1^. Various difficulties have so far hindered the translation of gene-targeted therapeutics from the lab into the clinic. The major obstacle is bypassing the plasma membrane to deliver the nucleic acid cargo to the intracellular target sites. Numerous genetic diseases are associated with mutations arising from aberrant alternative splicing, the essential mechanism to increase the complexity of gene expression. A very promising gene therapy approach for the modulation of splicing is the use of splice-correcting oligonucleotides (SCOs), which bind and restore the splicing of the pre-mRNA. SCOs are anti-sense oligonucleotides from 5 to 25 bases in length and can redirect splicing of a target pre-mRNA, used for example as a central modulator of several types of muscular dystrophies. In contrast with the traditional anti-sense approach, it must not activate RNase H, which in turn would destroy the pre-mRNA. To increase the stability SCOs contain chemical modifications compared to DNA or RNA.

Cell-penetrating peptides (CPPs) are short cationic peptides that have the capability of delivering cargos across cellular membranes with low toxicity^2–4^. The uptake pathways of CPPs are not entirely understood, and even less is known about the cellular responses and intracellular trafficking of CPP-cargo constructs.

Autophagy, Greek for “self-eating,” was discovered about 50 years ago, a discovery that was recently awarded the Nobel Prize in Physiology or Medicine. It is an evolutionarily conserved pathway in yeast, plants, worms, flies, and mammals. Autophagy is a pathway where a portion of the cytoplasm is isolated inside a double membrane vesicle, called autophagosome, that sequentially fuses with the lysosome for degradation^5^. It is one of the critical pathways for sustaining cellular integrity and homeostasis by degrading cytosolic molecules and defective organelles under natural physiological states. Moreover, autophagy is upregulated in response to stress, such as starvation, growth factor deprivation (therefore cells can recover fatty acids and amino acids to sustain metabolism for cell survival), hypoxia, oxidative stress, irradiation, and anti-cancer medications or intracellular infection of pathogens. In these cases, autophagy promotes stress adaptation and supports cell survival^6–9^. There is a significant and increasing number of research data showing that autophagy dysfunction is broadly associated with the progress of several degenerative disorders, including neurodegeneration^8,10–13^.

Autophagy mechanisms can be further classified into three principal classes, macroautophagy, microautophagy, and chaperone-mediated autophagy (CMA). Microautophagy comprises straight sequestration of gross cytoplasm or organelles inside the lysosomal lumen by septation, invagination, or projection of the lysosomal membrane.

The molecular mechanism of macroautophagy is well defined. The structural characteristic in macroautophagy is the formation of the autophagic vacuole, a process that can be divided into two sequential steps. First, the formation of autophagosomes, which arise with the phagophore generation (i.e. insulation membrane), next accompanied by elongation and cessation processes that drive to completion of a double-membrane-delimited vesicle. Second, a consequent fusion of the vesicles with lysosomes to develop autolysosomes, which contain lysosomal hydrolases and are surrounded by a single membrane^14^. The precise membrane origin of autophagosomes is still ambiguous, despite mitochondrial outer membrane, endoplasmic reticulum (ER) membrane, and plasma membranes have been sequentially claimed as their possible origin^15^. Distinctly, it has been proposed that the ER-mitochondria association loci are required in phagophore construction^16^. In the development of autophagosome, the cytoplasmic cargos can be randomly included in the autophagosome or selectively identified and isolated by the autophagic mechanisms. Degradation of the segregated cargos starts immediately upon autolysosome formation, and by that lysosomal hydrolases get access to those substrates^17^.

In CMA, lysosomes degrade the substrate proteins by recognizing a penta-peptide motif related to KFERQ. Distinctly, this motif is recognised based on the charge of amino acid residues and hydrophobicity. Instead of 100% agreement with particular amino acid residues, which are recognized specifically by HSP70 the cytosolic chaperone that delivers the proteins containing the identified motif to the exterior of the lysosomes. Already beyond, the chaperone complex with substrate binds to the cytosolic tail of lysosome-associated membrane protein type 2A (LAMP-2A) which functions as a receptor for managing unfolding and translocation of the substrate within the lysosomal lumen^18^.

The uptake process of CPP non-covalent nano-complexes with splice correction oligo (PF14/SCO nano-complexes) is fast, so the RNA expression pattern reflects the sub-cellular responses triggered by the treatment. Here, we investigated the changes in gene expression profiles occurring during transfection of HeLa cells by using RNA sequencing analysis and related them to cellular pathways that might be regulating nucleic acid transfection. We then verified the regulation of identified genes by transfection studies performed in the presence and absence of small molecules ligands for the proteins coded by these genes. We demonstrate the activation of autophagy pathway upon the cellular uptake of PF14 and its cargo. This analysis reveals that autophagy pathway governs the efficacy of nucleic acid transfection.

## Materials and Methods

### Reagents

Coupling reagents and amino acids were bought from Iris Biotech, Germany. Rink-amide ChemMatrix resin was purchased from PCAS BioMatrix, Canada. PS-2′-OMe splice-correcting oligonucleotides (5′-CCU CUU ACC UCA GUU ACA-3′) were purchased from RiboTask, Denmark. Cell culture reagents were purchased from Gibco (Life Technologies). Cyto-ID autophagy detection kit was bought from Enzo Life Sciences, Sweden. RNA extraction has been performed with RNeasy Mini Kit purchased from Qiagen, Germany and RNA Clean & Concentrator purchased from Biosite, Sweden. cDNA samples were prepared using RevertAid Minus First Strand cDNA Synthesis Kit purchased from Thermo Scientific, Sweden. All PCR reagents (primers and dyes) were purchased from Biorad, Sweden. Pharmacological ligands (CCG 203971, PF 573228, Atorvastatin LDL, Prostaglandin E2, TLR-4-IN-C34, CH-223191, Wortmannin, Bafilomycin A (BAFA), Rapamycin (RAP), and Importazole were from Sigma-Aldrich (Stockholm, Sweden); (MHY1485, Alprenolol hydrochloride) was purchased from Tocris Bioscience, Sweden.

### Peptide synthesis

PF14 (Stearyl-AGYLLGKLLOOLAAAALOOLL-NH_2_) was synthesized on a Rink Amide ChemMatrix resin (PCAS Biomatrix, Canada), by using a Biotage Initiator + Alstra microwave peptide synthesizer (Biotage, Sweden). N, N′-diisopropylcarbodiimide (DIC) and OxymaPure were the coupling reagents, and conventional Fmoc protected amino acid monomers were used (Iris Biotech, Germany). The peptide was cleaved by using 95% trifluoroacetic acid (TFA, Iris Biotech), 2.5% H_2_O, and 2.5% triisopropyl silane (Sigma–Aldrich) and was precipitated in cold diethyl ether. The collected crude peptide was suspended in 5% acetic acid and lyophilized. Reverse phase high-performance liquid chromatography (RP-HPLC) was used for peptide purification through a preparative BioBasic C8 column (ThermoFisher, Sweden) with a gradient of acetonitrile and water. The identity of the purified product was confirmed by using matrix-assisted-laser-desorption-ionization-mass spectrometry (Voyager-DE STR, Applied Biosystems). After RP-HPLC purification, the peptide was lyophilized, and the peptide solutions ﻿used following were dilutions of freshly dissolved peptides﻿.

### Cell cultures

HeLa and HeLa pLuc/705 cells were both cultivated in Dulbecco’s modified Eagle’s medium (DMEM, Life Technologies). DMEM was supplemented with 1 mM sodium pyruvate, glutamate, 10% fetal bovine serum (FBS, Life Technologies), 100 μg/ml streptomycin, with 100 U/ml penicillin, in a water-jacketed incubator at 37 °C and 5% CO_2_.

For the RNA levels experiments, 5 × 10^5^ HeLa cells were seeded into TC-treated 6-well plates (Corning) 24 h before treatment. On the day of treatment, the cell culture medium was replaced with two ml of new FBS-free media before treatment with peptides at 2 µM final concentration or complexes of peptide/oligonucleotide at a molar ratio (MR) 10. Cells were treated for 1 h and then harvested.

In the pharmacological ligands experiments, the splice-correcting assay was performed by seeding 7 × 10^3^ cells into tissue culture-treated, white, clear flat-bottom 96-well plates (Corning) 24 h before treatment. Then, the medium was changed with medium containing the pharmacological ligands in serial dilutions and incubated for 1 h before the peptide/oligonucleotide complexes transfection. Subsequent 24 h treatment, the utmost of the cell medium was accurately aspirated, and the plates were then centrifuged upside down at 300 rpm for 15 s in a plate centrifuge to empty them from cell medium. Then freeze (−80 °C)-thaw cycle was used to lyse the cells; then the frozen plates were left on the bench to reach room temperature (RT) before the measurements of luciferase activity.

For starvation-induced autophagy experiments, HeLa pLuc/705 cells were starved for 4 h in Hank’s Balanced Salt Solution medium (HBSS); then the treatment started for 24 h in full DMEM medium.

### RNA extraction

After 1 h treatment with the peptide or peptide/oligonucleotide, the media was aspirated, and the cells were harvested by trypsinization before resuspension in 2 ml media. The suspension was transferred to a 15 mL tube and centrifuged at 300 rpm for 5 min. Media was then aspirated, and the cell pellet was frozen at −80 °C. The RNA extraction was performed by using RNeasy kit. The RNA samples have shown a good purity according to A_260/280_ and A_260/230_ ratios. A portion of the samples was kept for RNA sequencing, and the rest was then converted to cDNA sample using a RevertAid Minus First Strand cDNA Synthesis Kit with random hexamer primers.

### RNA sequencing and analysis

The quality of purified total RNA was estimated by Agilent 2200 TapeStation analysis (Agilent Technologies, Santa Clara, USA). 1 µg of total RNA was used as an input to prepare next-generation sequencing libraries according to the Illumina TruSeq Stranded mRNA sample preparation protocol (Illumina, San Diego, USA). Final library mixtures were quantified by Qubit 2.0 Fluorometer (Life Technologies, Grand Island, USA) and validated with Agilent 2200 TapeStation analysis. Libraries were quantified by qPCR with Kapa Library Quantification Kit (Kapa Biosystems, Woburn, USA) to optimize cluster generation and sequenced on HiSeq 2500 platform (Illumina, San Diego, USA) with 2 × 50 bp paired-end reads. Over 93.9% of the bases sequenced were above the quality of Q30. Demultiplexing was done with CASAVA 1.8.2. (Illumina, San Diego, USA) allowing one mismatch in 6 bp index read. Initial data analysis was conducted by the RNA-Seq pipeline of Estonian Genome Centre, University of Tartu. Shortly, fastQ files were trimmed (removal of adapter sequences and bases below the quality Q20) with FASTX-Toolkit version 0.013 (http://hannonlab.cshl.edu/fastx_toolkit) and then aligned to the human reference genome (hg19/GRCh37) with Bowtie version 2.1.0^19^ in combination with TopHat version 2.0.13^20^. Transcript quantification (measured as FPKM) was conducted with Cuffdiff program from Cufflinks version 2.2.1^21^ with reference annotation Homo_sapiens.GRCh37.72.gtf (http://ftp.ensembl.org/pub/release-72/gtf/homo_sapiens) Cuffdiff analysis, which summarizes expression changes for all annotated gene variations, was filtered by lowest q-values (corrected p-values for multiple testing) from output file gene_exp.diff and the top list of differentially expressed genes were analyzed through the use of QIAGEN’s Ingenuity® Pathway Analysis (IPA®, QIAGEN Redwood City, www.qiagen.com/ingenuity).

### qPCR analysis

PCR primers were bought lyophilized in 96-well plates. In each well was added and mixed 100 ng cDNA in 10 µl DNase-free water. 10 µl of SsoAdvanced™ Universal SYBR® Green Supermix were also added to each well. A Biorad iQ5 Real-Time PCR Detection System was used to run the temperature cycles: 1 cycle of 2 min at 95 °C for activation, 40 cycles of 5 s at 95 °C for denaturation, 40 cycles of 30 s at 60 °C for annealing and finally 1 cycle with a temperature gradient from 65 °C to 95 °C with 0,5 °C increment every 5 s. SYBR green probe was used as a tracker to detect the cycle threshold of gene amplification. In addition to the genes of interest, 14 reference genes were tested using the same protocol. The values of cycle thresholds for these genes were used as reference cycle threshold for the calculation of fold change in the genes of interest expression.

### *In vitro* transfection

PF14 was dissolved in ultrapure water (MilliQ) at 1 mM, then aliquots of this solution were stored frozen at −20 °C. Aliquots were thawed on the day of treatment and diluted with ultrapure water to 100 μM. On the day of treatment, aliquots of the SCO were thawed and diluted to 10 μM. PF14 and SCO were mixed at a 10:1 MR in ultrapure water and were used in a final concentration of 200 nM SCO and 2 μM PF14.

The system for the SCO transfection assay is as follows. HeLa cells stably transfected with pLuc/705, a plasmid providing the firefly luciferase gene interrupted with human β-globin intron 2 displaying a cryptic splice site mutation, were used as a positive test system. If the wrong splice area is not hidden by SCOs, the pre-mRNA of luciferase will be inappropriately translated. SCO transfection drives to a raise in luminescence signal due to the correction of the splice site. The luciferase activity was measured using luciferase assay method (Promega) on Glomax 96 microplate luminometer (Promega).

The following primary antibodies were used: rabbit monoclonal anti-LC3B 1:500 [EPR18709] (ab192890) for immunofluorescence and 1:1000 for Western blot, mouse anti-β-actin 1:5000 (A 5441, Sigma). The secondary antibodies used for Western blotting were horseradish-peroxidase-coupled donkey anti-mouse IgG (NA931, GE healthcare), or donkey anti-rabbit IgG (NA934, GE healthcare). The secondary antibodies used for immunofluorescence was Alexa Fluor 488 goat anti-rabbit IgG (A11008, Invitrogen).

### Lysate preparation and Western blot analysis

HeLa pLuc/705 cells were untreated or treated with 500 nM RAP, 2 μM PF14, PF14/SCO nano-complexes with or without 500 nM Bafilomycin A (BAFA). HeLa pLuc/705 cells were washed with 1x PBS three times and scraped off by using rubber policeman. The cell pellets were collected by centrifugation at 800 g for 5 min and suspended in 1x sample buffer containing 100 mM DTT and boiled for 5 min at 95 °C. Equal amounts of whole cell lysates were loaded on 4–20% SDS-PAGE precast gels (Bio-Rad, #456–1095). SDS-PAGE separated proteins were transferred onto PVDF (Bio-Rad, #1060002) membranes, then blocked with 5% milk in PBS-T (blocking solution) for 1 h at RT. The membranes were incubated with primary antibodies in the blocking solution for 1 h at RT. Next, the membranes were washed 3 × 10 min in PBS-T, then the membranes were incubated with secondary antibody in the blocking solution for 1 h at RT. Following 4 × 10 min washes in PBS-T, the membranes were subjected to ECL detection (Amersham ECL prime, RPN2232, GE healthcare). The emitted chemiluminescent signal was examined by ChemiDoc XRS + imaging system (Bio-Rad).

### Staining of autophagosomes in live HeLa cells

HeLa pLuc/705 7 × 10^3^ cells were seeded in a 96-well plate with glass bottom 24 h before treatment. Cells were incubated in serum-containing media with/without ligand for 1 h; then the fluorescent PF14/SCO(Alexa568) complexes (MR10, 100 nM SCO) were applied and incubated with cells for 24 h. Cells were then stained with Cyto-ID kit (Enzo Life Sciences) according to the manufacturer’s instruction. Imaging was performed using Leica DM/IRBE 2 epi-fluorescence microscope controlled by Micro-Manager^19^ with a 63 × 1.4 NA oil immersion objective. Images were analyzed using Fiji (ImageJ) software^20^. Quantification was performed by acquiring a stack with 0.3 µm slices through the cell. The stack was filtered with the 3D mean filter using 2 × 2 × 2 setting. Cells were then manually marked as a region of interest (ROI) and the signal from the ROIs with the highest average intensity used. Cell-free ROIs next to the measured cell was used for background subtraction.

### Immunofluorescence

HeLa pLuc/705 7 × 10^3^ cells grown on 96-well glass bottom plate were rinsed two times with PBS, and fixed in methanol for 10 minutes at −20 °C. The cells were then permeabilized with 0.5% TX-100 in 1x PBS for 5 min on ice. Following three washes with PBS, the cells were blocked with 2% BSA in PBS-T (blocking solution) for 1 h and incubated with primary antibodies in blocking solution for 1 h. After 4 washes with blocking solution, the samples were incubated with secondary antibodies antibody containing Draq5 (1:5000) in blocking solution for 1 h. Following further washes with PBS-T, the glass-bottomed wells were imaged. Draq5 was used to stain the DNA. Also 1x PBS was used in all washing steps.

### Imaging and image processing for immunofluorescence

Imaging was performed by using a Leica DM/IRBE 2 epi-fluorescence microscope with a 63 × 1.4 NA oil immersion objective for fluorescence imaging, and images were recorded by a Hamamatsu Orca-ER CCD camera. The system was controlled by the Micro-Manager^19^. Fluorescent images were collected using GFP, N3, and ET CY5 filter cubes (Chroma Technology Corporation, VT, USA) for Alexa 488, Alexa 568 and Draq5 respectively. Micrographs were collected for cells expressing LC3B marker protein and assessed for total fluorescence. Cells were manually segmented and region intensities quantified using Fiji (ImageJ)^20^. To achieve an unbiased measure of the intensity of the SCO in the nuclei, we used automated image sampling and programmed determination of nuclear areas using the free image analysis software CellProfiler.

### Labelling of pDNA for mapping autophagy by electron microscopy

To analyze the induction of autophagy by the nano-complexes of PF14 with nucleic acid^22^, transmission electron microscopy (TEM) analysis was combined with the application of autophagy inducers and gold-tagged nucleic acid. For the visualization by TEM, the pGL3 plasmid was tagged with biotin and complexed with neutravidin-gold (NA-gold) conjugate (10 nm in diameter) (homemade)^23^. Biotin was covalently coupled to pDNA by using Nucleic Acid Labelling Kit according to manufacturer’s protocol (Label It Nucleic Acid Labelling Kit, Biotin, Mirus Bio, USA) as described earlier^24^. Biotinylated pDNA (1 μg/ml in MilliQ water) was first associated with NA-gold conjugate at 0.53 nM concentration for 30 min at 37 °C followed by complexing with PF14 at CR2 in MilliQ water in 100 μl (10% of the final treatment volume) for 30 min at room temperature.

### Treatment of cells for electron microscopy

HeLa cells were seeded onto glass coverslips in 35-mm Petri dishes, grown to 90% confluence and incubated with full culture medium containing PF14 complexes with pDNA-NA-gold for 1, 4, 8 or 24 h at 37 °C. After 4 h the medium that contained complexes was removed and replaced with fresh medium. As a positive control of autophagy induction, the cells were incubated with rapamycin (0.5 µM) or tamoxifen (5 μM) for the same time or were left untreated. After incubation, the coverslips with cells were placed in 24-well plates, washed, and fixed with 2.5% glutaraldehyde in cacodylate buffer (pH 7.4) for 1 h at ambient temperature. The cells were post-fixed with osmium tetroxide and dehydrated with ethanol.

The specimens were embedded in epoxy resin (TAAB Laboratories Equipment Ltd., UK), cut into ultrathin sections and contrasted with uranyl acetate and lead citrate^25^. The sections were examined with Tecnai G2 SpiritTM (FEI, Eindhoven, Netherlands) transmission electron microscope and microphotos were analyzed and processed with Adobe Photoshop software^26^.

### Molecular docking of PF14 peptide backbone with HSP70 protein targets

Solution X-RAY diffraction structure of Polypeptide-Binding Site in a Human HSP70 Chaperone BiP (PDB ID: 5E85)^27^ was obtained from Protein Data Bank (PDB) (http://www.rcsb.org/pdb). PF14 peptide backbone was used for peptide-protein interaction investigation with HSP70 Polypeptide-Binding Site. The docking analysis was done by using GalaxyPepDock tool^28^.

### Data availability

RNA sequencing data, which we generated and investigated in the current study, are available at GEO database with accession code GSE92478 (https://www.ncbi.nlm.nih.gov/geo/query/acc.cgi?token=qlkjyqgkvbejzcj&acc=GSE92478).

### Statistical analysis

Data generated from 3 independent experiments in triplicates were statistically analyzed by one-way ANOVA test using GraphPad prism.

## Results

### RNA sequencing

**Figure 1:**
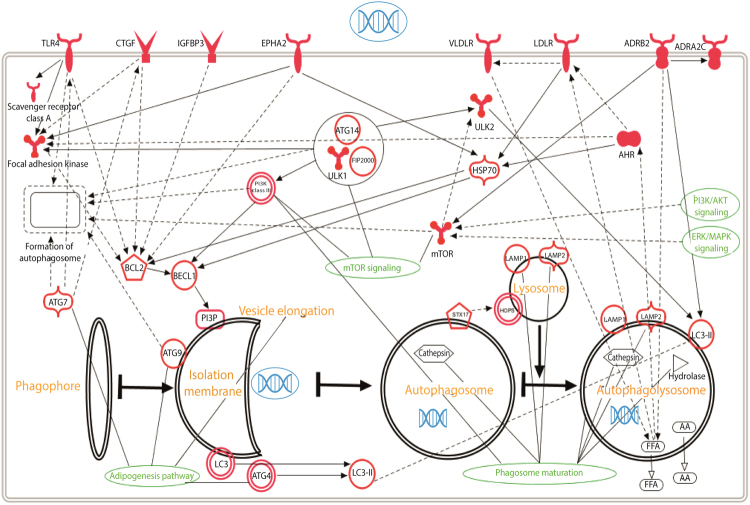
Autophagy pathway induced by transfection with PF14.

We performed RNA sequencing to study the effects of CPPs on the gene expression of HeLa cells. We used PF14 as a CPP and compared the pattern of gene expression in cells treated with CPP to that of untreated cells. Additionally, we tested the effect of cargo molecule on the gene expression when comparing the pattern of gene expression in cells treated with PF14 plus cargo (PF14/SCO) to that of untreated cells and cells treated with PF14. We used Cuffdiff analysis to summarize expression changes for all the annotated gene variations. The data were filtered by the lowest q-value and genes with significant expression changes (q < 0.01) are presented in Supplementary Tables 1 and 2. Our RNA sequencing results showed the most significant genes’ expression changes in case of treatment with PF14. Namely, there were 292 genes differentially expressed in PF14 treatment and 934 genes in PF14/SCO cargo. Next, we used IPA analysis to group genes with significant expression changes in pathways. According to the results of IPA analysis, PF14 has affected multiple pathways (Fig. 1). Following RNA sequencing, we chose to validate several of the most differentially expressed genes with qPCR. The genes were selected based on their potential to regulate autophagy due to the possible involvement of autophagy pathway in the regulation of the transfection process. Usually ULK1 functions in a complex with ATG13, ATG101 and FIP2000 but interestingly none of those three proteins have been shown to be regulated by the uptake of PF14 nor PF14/SCO. The same applies for ATG14 and its interaction partner within the hVps34 complex.

### qPCR validation

**Figure 2:**
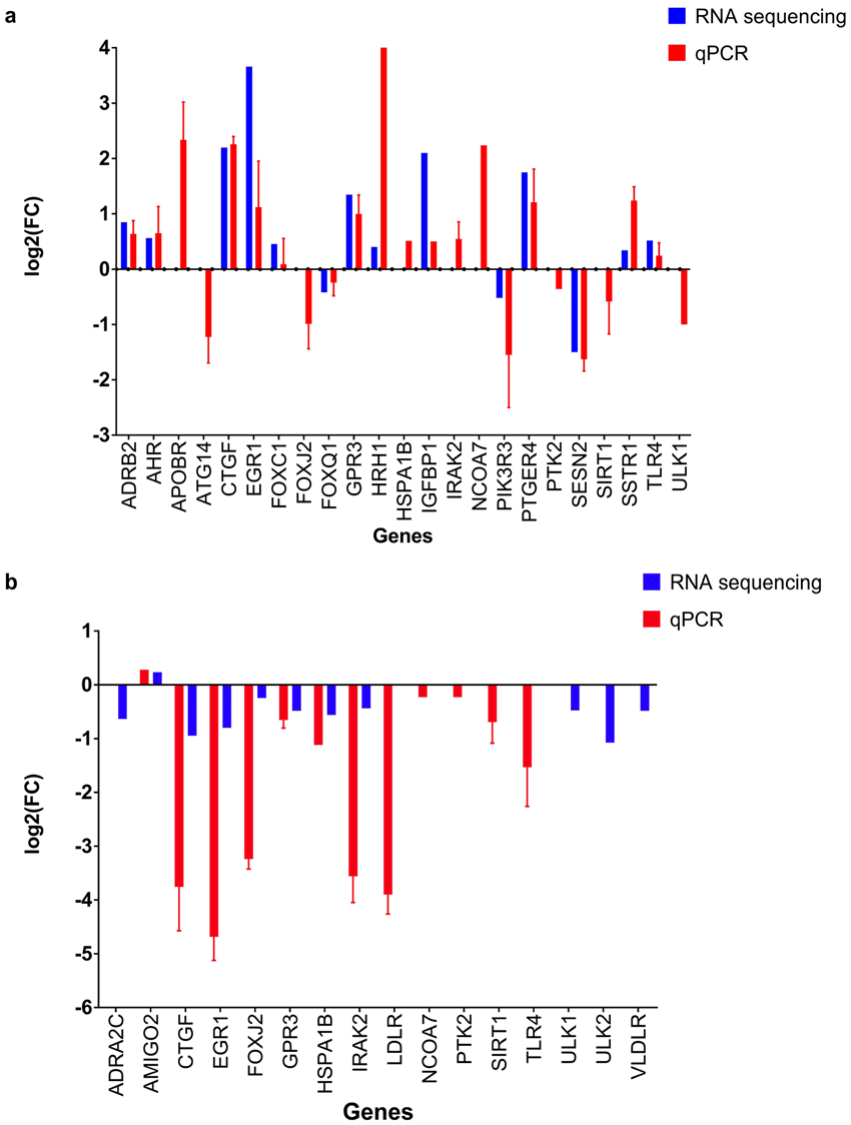
PF14 induced changes in the transcriptome of HeLa cells. The most relevant changes in gene expression are expressed as log_2_ of fold change as estimated by qPCR and RNA sequencing. RNA sequencing and qPCR analysis on RNA extracted from HeLa cells after 1 h treatment with PF14 (**a**) or PF14 and splice correction oligo (PF14/SCO) nano-complexes (**b**). The cycle threshold obtained from qPCR has been transformed into fold change by using a set of references genes (q < 0.01).

The qPCR measurements allowed to estimate the cycle threshold of gene amplification. These cycle thresholds were then analyzed using the cycle threshold of the reference genes and compared to untreated samples. Expression of 26 autophagy-related genes was analyzed (Adrenoceptor Beta 2 (ADRB2), Apolipoprotein B Receptor (APOBR), Connective Tissue Growth Factor (CTGF), Toll-Like Receptor 4 (TLR4), Forkhead Box C1 (FOXC1), Forkhead Box Q1 (FOXQ1), Interleukin 1 Receptor Associated Kinase 2 (IRAK2), Unc-51 Like Autophagy Activating Kinase 1 (ULK1), Sestrin 2 (SESN2), Somatostatin Receptor 1 (SSTR1), Histamine Receptor H1 (HRH1), Aryl Hydrocarbon Receptor (AHR), Autophagy Related 14 (ATG14), Nerve Growth Factor-Induced Protein A (EGR1), G Protein-Coupled Receptor 3 (GPR3), Forkhead Box J2 (FOXJ2), Insulin-Like Growth Factor Binding Protein 1 (IGFBP1), Low-Density Lipoprotein Receptor (LDLR), Phosphoinositide-3-Kinase Regulatory Subunit 3 (PIK3R3), Prostaglandin E Receptor 4 (PTGER4), Adhesion Molecule With Ig-Like Domain 2 (AMIGO2), Ephrin Type-A Receptor 2 (EPHA2), Heat Shock Protein Family A (Hsp70) Member 1B (HSPA1B), Nuclear Receptor Coactivator 7 (NCOA7), and Focal Adhesion Kinase-Related Non-kinase (PTK2)). The results of the analysis are shown in Fig. 2, expressed as a logarithm base 2 of the fold change in gene expression between treated and untreated samples. High fold changes in the gene expression were detected for several genes. For example, during PF14 uptake CTGF, PTK2, IGFBP1, PTGER4, HRH1, NCOA7 and APOBR expression was upregulated by at least 2 times and even more for some of the genes (4 times fold change for CTGF, NCOA7, APOBR) whereas PIK3R3, SESN2, ULK1, FOXJ2, and ATG14 showed a downregulation by at least 0,5 fold changes. LDLR expression showed a noteworthy down-regulation by 10 times. For PF14/SCO uptake experiments, we obtained a completely different profile with mostly down-regulation of the autophagy-related genes. These differences in the uptake regulation profile can be explained by the structural differences between nanoparticle formed by positively charged molecule and the complex formed by positively and negatively charged molecules. Although we still observed a ten times down-regulation of LDLR expression, we saw CTGF, IRAK2, EGR1 and FOXJ2 expressions presented a similar down-regulation as well. TLR4, GPR3, PIK3R3 and HSPA1B expressions were also down-regulated by the uptake of our complex between 0,25 and 0,5 fold change. Analogous effects were also detected on a proteome level in previous research on PepFect 6. Indeed the ATG, HSP, and FOX families have been shown to be influenced by the presence of PepFect 6^29^.

### Autophagy-regulating ligands

**Figure 3:**
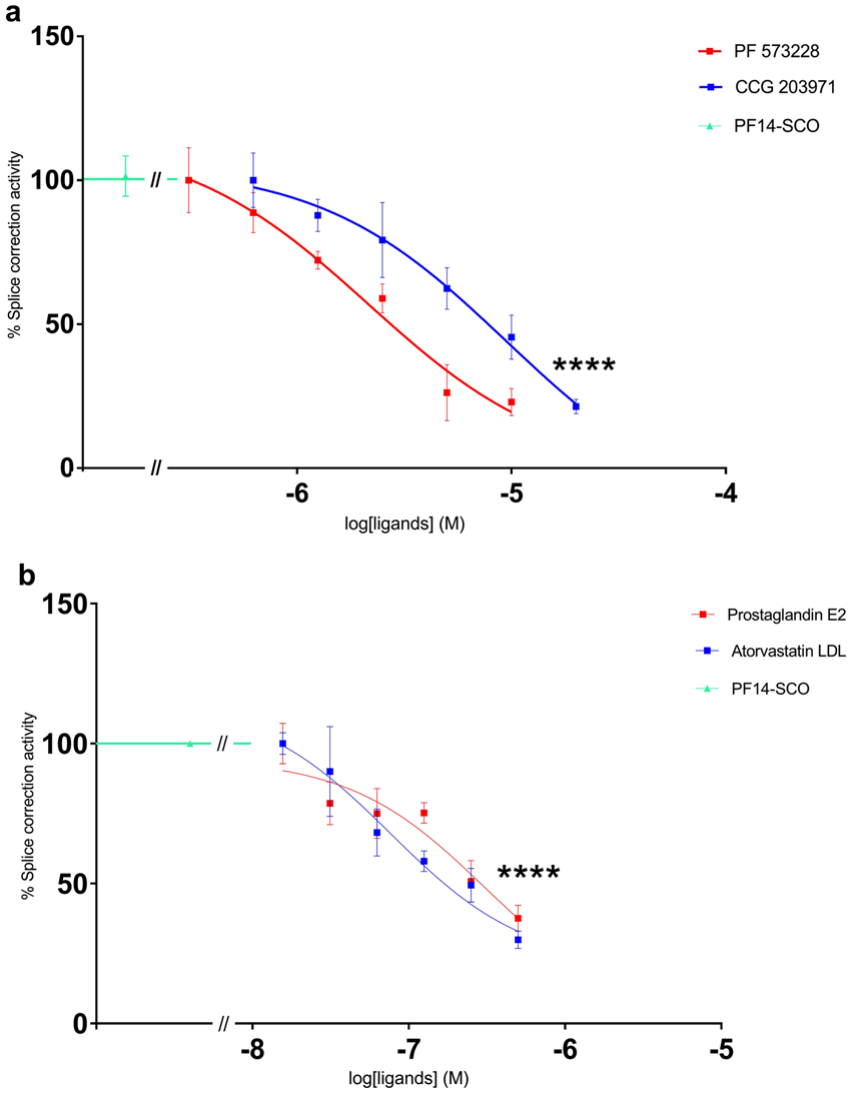
Downregulation of splice correction activity in a concentration-dependent manner by using small molecule ligands. (**a**,**b**) HeLa pLuc/705 cells treated for 24 h with PF14 and splice correction oligo (PF14/SCO) nano-complexes and splice correction suppressing ligands [CCG 203971 (CTGF inhibitor), PF 573228 (focal adhesion kinase (FAK) inhibitor), Atorvastatin LDL (HMG-CoA reductase inhibitor), Prostaglandin E2]. Splice correction activity is based on luminescence signal from splice corrected luciferase. Splice correction activity was normalized to the baseline signal of untreated cells, and 100% corresponds to treatment with PF14/SCO complexes (P < 0.0001).

**Figure 4:**
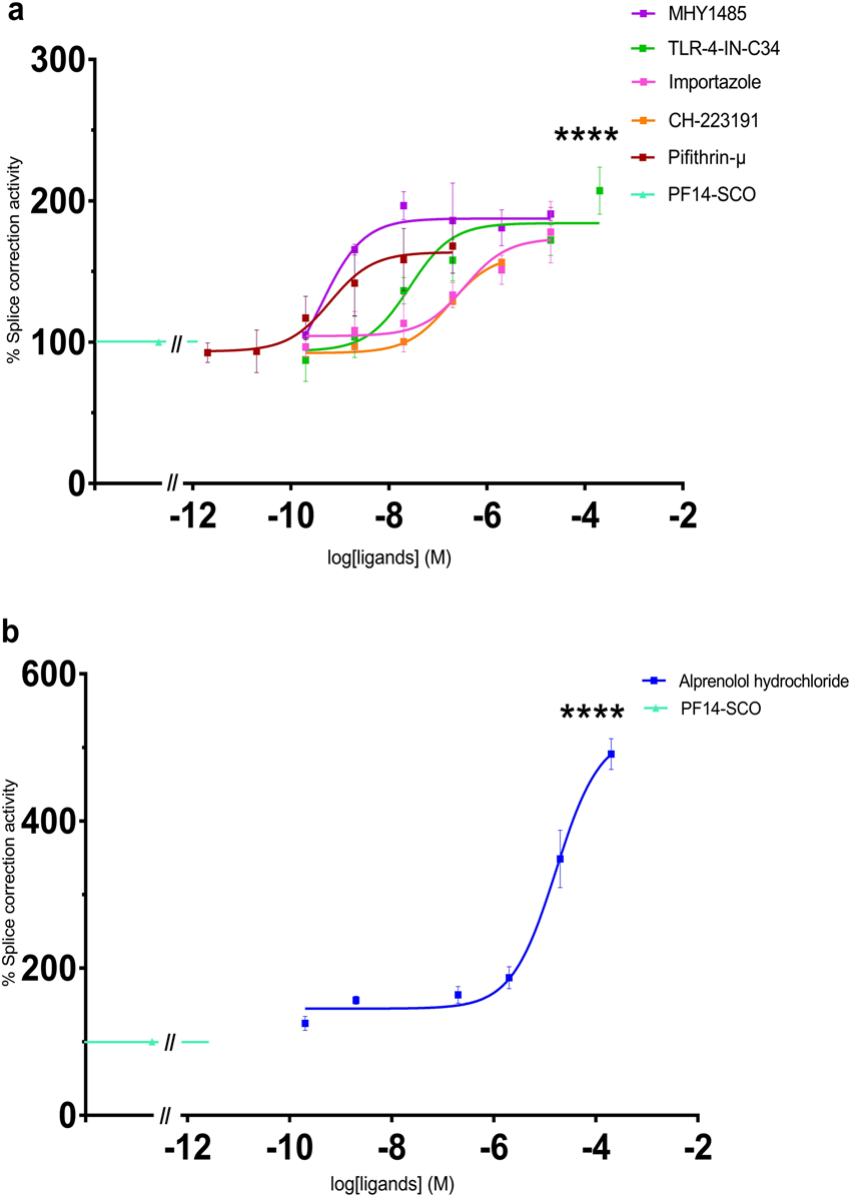
Upregulation of splice correction activity in concentration-dependent manner by using small molecule ligands. (**a**,**b**) HeLa pLuc/705 cells treated for 24 h with PF14 and splice correction oligo (PF14/SCO) nano-complexes and splice correction activating ligands [Pifithrin-μ (selective inhibitor of heat shock protein 70 (HSP70)), Alprenolol hydrochloride (β-adrenoceptor antagonist), CH-223191 (aryl hydrocarbon receptor (AHR) antagonist), Importazole (nuclear transport receptor importin-β inhibitor), TLR-4-IN-C34 (TLR4 inhibitor), MHY1485 (mTOR activator)]. Splice correction activity is based on luminescence signal from splice corrected luciferase. Splice correction activity was normalized to the baseline signal of untreated cells, and 100% corresponds to PF14/SCO complexes treatment (P < 0.0001).

Yang *et al*. demonstrated that it is feasible to use high-throughput small molecules library screening to determine small molecules that actively improve the pharmacological effects of oligonucleotides^30^. PF14 in complex with the SCO was used together with ligands that modulate autophagy pathway, which are known to activate or inhibit particular proteins in the pathway. The proteins were chosen based on the selected genes from the pathway analysis, used as a screening tool to assess whether a particular gene may play a significant role in the uptake mechanism. This selection included proteins having a potential to regulate autophagy based on the pathway analysis of the RNA sequencing data. Cells were incubated with ligands for 1 h, and then PF14/SCO complexes were added. Luciferase activity was measured 24 h later. The concentration-dependent effects of the ligands on splice correction levels are summarized in Tables 1 and 2 with luciferase splice correction levels provided in Figs 3 and 4. In the case of all ligands reported here, little-to-no cytotoxicity was observed at the highest concentrations used.

**Table 1:** Characterization of the downregulation in splice correction activity induced by small molecule ligands.

Splice correction Inhibition ligands	IC_50_ (nM) ± SEM
CCG 203971 (CTGF inhibitor)	5020 ± 290
PF 573228 (focal adhesion kinase (FAK) inhibitor)	5670 ± 240
Atorvastatin LDL (HMG-CoA reductase inhibitor)	71 ± 0.25
Prostaglandin E2	651 ± 0.41

CCG 203971“*N*-(4-Chlorophenyl)-1-[3-(2-furanyl)benzoyl]-3-piperidinecarboxamide”, PF 573228“ 3,4-Dihydro-6-[[4-[[[3-(methylsulfonyl)phenyl]methyl]amino]-5-(trifluoromethyl)-2-pyrimidinyl]amino]-2(1 *H*)-quinolinone”, Atorvastatin LDL“(*R*,*R*)-2-(4-Fluorophenyl)-β,δ-dihydroxy-5-(1-methylethyl)-3-phenyl-4-[(phenylamino)carbonyl]-1*H*-pyrrole-1-heptanoic acid hemicalcium salt” and Prostaglandin E2“(5*Z*,11α,13*E*,15 *S*)-11,15-Dihydroxy-9-oxo-prosta-5,13-dien-1oic acid”.

**Table 2:** Characterization of the upregulation in splice correction activity induced by small molecule ligands.

Splice correction activation ligands	EC_50_ (nM) ± SEM
Pifithrin-μ (selective inhibitor of heat shock protein 70 (HSP70))	0.92 ± 0.51
Alprenolol hydrochloride (β-adrenoceptor antagonist)	47000 ± 120
CH-223191 (aryl hydrocarbon receptor (AhR) antagonist)	676 ± 0.26
Importazole (nuclear transport receptor importin-β inhibitor)	650 ± 460
TLR-4-IN-C34 (TLR4 inhibitor)	76 ± 0.32
MHY1485 (mTOR activator)	9.39 ± 0.61

Pifithrin-μ “2-Phenylethynesulfonamide”, Alprenolol hydrochloride “1-(o-Allylphenoxy)-3-(isopropylamino)-2-propanol hydrochloride“, CH-223191 “1-Methyl-*N*-[2-methyl-4-[2-(2-methylphenyl)diazenyl]phenyl-1*H*-pyrazole-5-carboxamide”, Importazole “N-(1-Phenylethyl)-2-(pyrrolidin-1-yl)quinazolin-4-amine”, TLR-4-IN-C34 “2-Acetamidopyranoside, C34, Isopropyl 2-(acetylamino)-2-deoxy α-D-glucopyranoside 3,4,6-triacetate” and MHY1485 “4,6-Di-4-morpholinyl-N-(4-nitrophenyl)-1,3,5-triazin-2-amine”.

**Figure 5:**
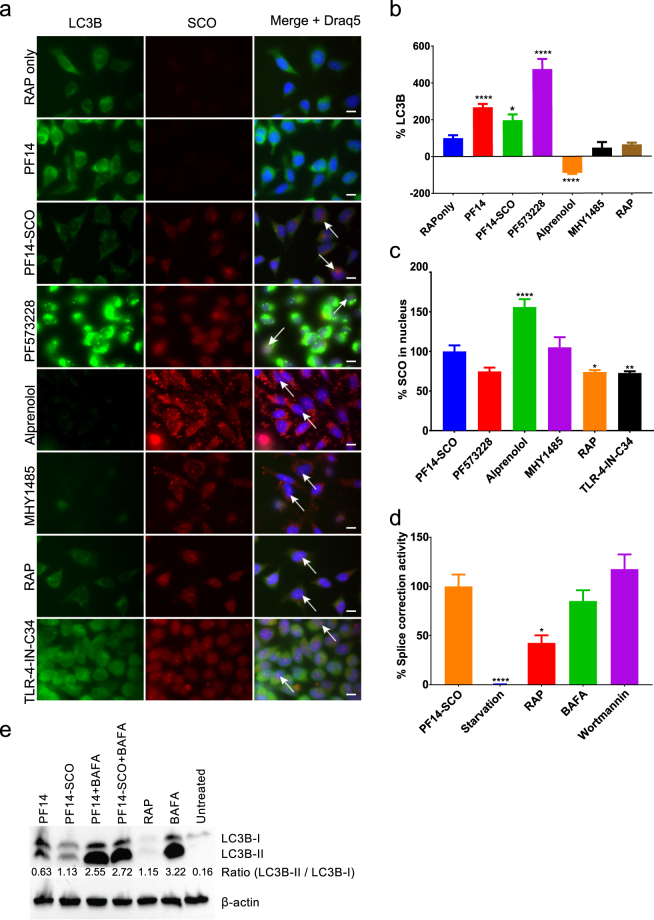
Induction of autophagy by PF14 and its complexes with SCO. (**a**) Epifluorescence micrographs of HeLa pLuc/705 cells treated for 24 h with Rapamycin (RAP) 500 nM, or 2 μM PF14, or PF14 and splice correction oligo labelled with Alexa 568 nano-complexes (PF14/SCOAlexa568) (red, formed at molar ratio 10/1) with or without 6 µM focal adhesion kinase inhibitor (PF573228), 10 µM Alprenolol hydrochloride (β-adrenoceptor antagonist), 10 nM mTOR activator (MHY1485), 500 nM RAP, 76 nM TLR-4-IN-C34 (TLR4 inhibitor). Immunofluorescence of LC3B as autophagy marker protein is shown (green), arrows point to SCO colocalized with the nucleus. (**b**) Quantification of green immunofluorescence signal of LC3B is with the same treatments as in (**a**). Data were normalized to the baseline green signal of untreated cells, and the 100% as a signal from treatment with RAP alone without PF14/SCO complexes. (**c**) Quantification of the red signal from PF14/SCO(Alexa568) complexes in the nucleus in the treatments of (**a**) which have the complexes, data was normalized to the baseline red signal of untreated cells, and the 100% is PF14/SCO(Alexa568) complexes. (**d**) HeLa pLuc/705 cells treated with PF14/SCO complexes with or without starvation for 4 h with EPSS medium, 500 nM RAP, 500 nM Bafilomycin A (BAFA), 500 nM Wortmannin for 24 h in full DMEM medium. Splice correction activity is based on luminescence signal from splice corrected luciferase. Splice correction activity was normalized to the baseline signal of untreated cells, and the 100% is PF14/SCO complexes treatment. (**e**) Western blot of LC3B for HeLa pLuc/705 cells lysates, the cells were untreated or treated with 500 nM RAP, PF14, PF14/SCO nano-complexes with or without 500 nM BAFA. LC3BII/LC3BI protein ratios were calculated from band intensities normalized to β-actin bands intensities. Scale bar is 10 µm (*P < 0.0351,**P < 0.0033, ****P < 0.0001).

Splice correction was significantly (P < 0.0001) reduced by ligands presented in Fig. 3 and Table 1, e.g. CTGF inhibitor and focal adhesion kinase (FAK) inhibitor reduced 75% of the splice correction activity (Fig. 3a). Also, HMG-CoA reductase inhibitor and Prostaglandin E2 inhibited the splice correction activity by 60% (Fig. 3b). On the other hand, splice correction efficiency was significantly (P < 0.0001) improved with ligands, shown in Fig. 4 and Table 2, as measured by luciferase activity. Additionally, mTOR activator, TLR4 inhibitor, and the nuclear transport receptor importin-β inhibitor increased the splice correction activity (Fig. 4a). Furthermore, aryl hydrocarbon receptor (AhR) antagonist and the inhibitor of heat shock protein 70 (HSP70) increased the splice correction by 1.5 fold, and the β-adrenoceptor antagonist upregulated the splice correction activity by 5-fold (Fig. 4b). Interestingly, starvation significantly inhibited the splice correction activity (Fig. 5d).

### Detection and quantification of autophagy in HeLa cells with confocal, fluorescent and electron microscopy

The LC3B antibody and Cyto-ID signals were detected in cells treated with PF14/SCO(Alexa568) for 8 h, but these were not present after 1 h incubation (data not shown). The staining was even more intense after 24 h incubation (Supplementary Figure 1). Localization of PF14/SCO(Alexa568) complexes fluorescence signal largely overlapped with stained autophagosomes as analyzed by CLSM (Supplementary Figure 1, yellow signal). The colocalization rates were 88% for PF14/SCO(Alexa568)/autophagosomes; treatment with mTOR activator (MHY1485) reduced it to 68% (Fig. 5a,b, Supplementary Figure 1d) and FAK inhibitor (PF573228) to 80% (Supplementary Figure 1c). This indicates that most of the PF14/SCO(Alexa568) complexes end up in Cyto-ID stained organelles. However, when autophagosome formation is induced by pharmacological inhibitors, the LC3B antibody and Cyto-ID stained vesicles that do not contain SCO also emerge. This is further supported by the quantification of the LC3B antibody and Cyto-ID signals that show an increase after treatment with PF14/SCO(Alexa568) (MR10) or PF14 alone at the same concentration compared to untreated cells (Fig. 5a,b, Supplementary Figure 1e). The Cyto ID staining intensity increased even further upon the coincubation of PF14/SCO(Alexa568) with either 10 nM of mTOR activator (MHY1485), 6 µM of FAK inhibitor (PF573228), or 76 nM of TLR4 inhibitor (TLR-4-IN-C34) (increased LC3B antibody signal also) that both trigger accumulation of autophagosomes (Fig. 5a,b, Supplementary Figure 1).

### Influence of autophagy-regulating ligands on cellular uptake of PF14/SCO(Alexa568)

The cellular uptake levels of PF14/SCO(Alexa568) were observed and quantified by measuring intracellular PF14/SCO(Alexa568) red signal along with nucleus staining Draq5 (Fig. 5a,c). The average cellular uptake of the PF14/SCO(Alexa568) complexes in HeLa pLuc/705 cells was used as highest fluorescence signal (100%), while untreated cells were used as the baseline for data normalization. By alprenolol treatment, the average cellular uptake of the PF14/SCO(Alexa568) significantly increased, by about 50% (P < 0.0001) as compared to nuclear localization of PF14/SCO(Alexa568) alone, which well correlates with the increased splice-correction activity in the presence of alprenolol shown in Fig. 4b. The influence of β-adrenoceptor regulation on the autophagy pathway is also supported by the significant (P < 0.0001) inhibition of autophagy, as shown in Fig. 5a,b, which also resulted in higher PF14/SCO(Alexa568) nuclear localization and splice correction activity (Figs 5a,c and 4b). On the other hand, RAP treatment, which is conventionally used as an autophagy inducer, reduced the PF14/SCO(Alexa568) nuclear localization and splice correction significantly (P < 0.03) (Fig. 5a,c,d).

**Figure 6:**
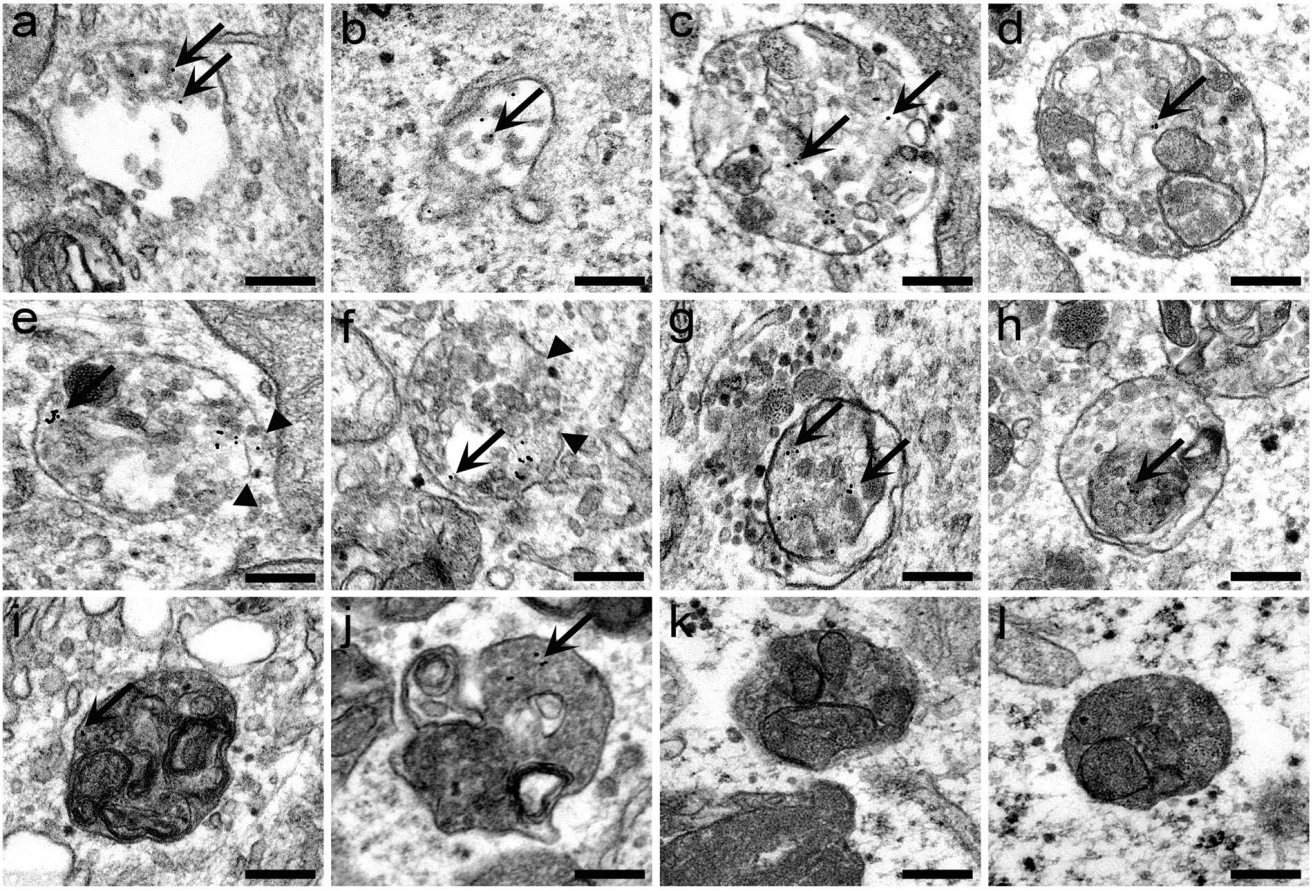
Analysis of the cellular uptake of PF14/pDNA complexes and induction of autophagy using transmission electron microscopy. HeLa cells were incubated with PF14/pDNA nano-complexes (0.1 μg/ml CR 2) tagged with 10 nm colloidal gold particle (black dots) for 1 h (**a**,**b**) 4 h (**c**–**f**), 8 h (**g**,**h**) or 24 h (**i**,**j**). Localization of PF14/pDNA complexes (pointed by arrows) in early (**a**,**b**) and late endosomal organelles (**c**–**f**); in autophagosomes (**g**,**h**) and autolysosomes (**i**,**j**). The control cells were treated with 0.5 μM rapamycin for 24 h to promote the formation of autophagosomes and autolysosomes (**k**,**l**). Arrows point to PF14/pDNA nano-complexes in the particular organelles, and arrowheads show the fragmentary regions in the limiting membrane of endosomes. Scale bar is 200 nm.

Analysis of autophagy induction using confocal microscopy revealed the recruitment of fluorescent PF14/SCO complexes to autophagosomes already after 8 h treatment in HeLa cells. To examine the cellular delivery of nucleic acids by PF14 in more detail, electron microscopy was used to better visualize them in the cells. It has been previously shown that PF14 trigger endocytosis to translocate it’s nucleic acid cargoes into cells^31–33^. Indeed, after 1 h incubation the gold labelled PF14 complexes with nucleic acid mainly localized to early endocytic structures (Fig. 6a,b, pointed by arrows), which possessed characteristic low electron density and lacked intraluminal vesicles. Later, the complexes were found in more matured organelles of the endolysosomal pathway, and after 4 h these were found mostly in late endosomes/multivesicular bodies with more condensed content and a high number of internal vesicles (Fig. 6c,d, pointed by arrows). Maturation and acidification of endosomal vesicles^34^ are necessary for the cargo molecules to escape from a particular subset of endosomes. In analogy with our report^35^ and earlier studies, after 4 h of uptake, endosomes with the discontinuous limiting membrane (Fig. 6e,f, arrowheads) appeared in cells, and the complexes escaped into the cytosol, which is a very rare event per se^36^. The interference with endosomes intactness induced by transfection reagent exposes the endosomal glycans to the cytosol, triggering recruitment of galectins and LC3^34^ and inducing macroautophagy^37^. Destabilization and disruption of the membrane of the endosome by PF14 nano-complexes also incurs autophagy, and the leaking endosomes are sequestered in autophagosomes (Fig. 6g,h). After fusion of autophagosomes with lysosomes, autolysosomes form, efficiently labeled by Cyto ID Tracker (Supplementary Figure 1) and LC3B antibody (Fig. 5a). The fate of PF14/pDNA in cells can be followed up, and they were found in autolysosomes tracking the gold tag incorporated in the complexes (Fig. 6i,j, arrows). The autophagosomes entrapping PF14/pDNA complexes might have very different morphology (Fig. 6i,j) but are reminiscent of organelles generated in cells by rapamycin (Fig. 6k,l) or tamoxifen (not shown), two known inducers of autophagy.

### Western blot of LC3B proteins

LC3B proteins are standard markers of autophagy induction, so we investigated their expression levels by Western blot. The generation of autophagosomes needs the LC3B-II protein, which is a product of the modification of the LC3B-I protein with phosphatidylethanolamine. The ratio of LC3B-II over LC3B-I protein levels is commonly used as an indicator of the autophagic induction and is correlated to the levels of the autophagosome formation^38^. LC3B-II/LC3B-I protein ratios were calculated from band intensities normalized to β-actin bands intensities. The LC3B-II/LC3B-I ratio was increased in HeLa cells treated with PF14 and PF14/SCO complexes as compared to untreated cells (Fig. 5e). This increase indicates that PF14 and PF14/SCO complexes are inducing autophagy in cells. Interestingly, the ability of PF14 to induce autophagy was comparable to cells treated with the standard autophagy inducer (Rapamycin).

### Possible PF14 interactions with HSP70 protein targets from in silico molecular docking study

**Figure 7:**
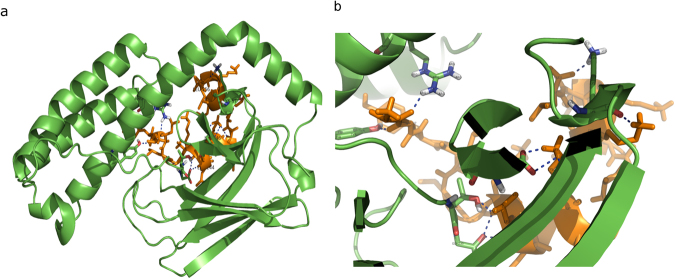
Molecular docking analysis of possible PF14 interactions with HSP70 protein. PF14 peptide is shown in orange, HSP70 is shown in green and hydrogen bonds are shown in blue dashes.

PF14 peptide backbone was found to have the possibility of interaction with HSP70 Polypeptide-Binding Site, as shown in Fig. 7a,b. The protein template used for this study was 1DKZ_A, and 1DKZ_B was used as peptide template. The protein structure similarity (TM-score) was found to be 0.837, interaction similarity score was 96.0, and the estimated accuracy was 0.681. Further understanding of this particular interaction in more detail is interesting for a separate investigation in a follow-up study.

## Discussion

We examined the intracellular trafficking of PF14 transfection system using a transcriptomics strategy aiming to unravel the pathways implicated in fundamental cellular phenomena associated with uptake of PF14 transfection system.

Autophagy is among of innate protection mechanism that is involved in degradation of invading pathogens, misfolded proteins, and dysfunctional cellular organelles. Autophagy is stimulated by nanoparticle delivery typically through pattern recognition receptors^39^. Here we report that TLR4 is associated with the autophagy induced by PF14 and it’s transfection complexes as we detected a significant increase of splice correction in case of TLR4 inhibition, in accordance with the known role of TLRs in the non-canonical recruitment of Atg proteins to a single-membrane bound phagosome and phagophore formation process^40^. It has been shown that nanoparticles are transferred into autophagosomes, efficiently confined of the encompassing cytoplasm, diminishing the connected toxicity and assisting in decreasing cellular tension^41^.

Induction of the autophagy mechanism has been earlier shown to accompany internalization of cationic polyplexes, lipoplexes, dendrimers, silica, iron oxide and gold nanoparticles^42–46^. In the current study, we show that cationic CPP, PF14 itself and in complex with nucleic acid cargo are also inducing autophagy upon cell transfection. Currently, the correlation between the nanoparticle surface characteristics and induction of the autophagic pathways seem to exist, but it is not clear why this route is possibly selective for the positively charged particles. Although autophagy may be advantageous for diminishing particles cytotoxicity, it can be deleterious for the delivery of cargo, as in the current case where capture of PF14 to autophagosomes reduces the splicing correction efficacy. If nucleic acid transduction system finally conveys cargoes to the autophagic organelles, only a tiny amount of the cargo is transferred to the target cellular destination, critically restricting efficiency and leading to oblige higher doses. This is in agreement with Roberts *et al*. finding, where they described that polyplex composed of plasmid DNA and a proprietary polymer (JetPRIME, composition unknown) were endocytosed and confined in cellular tubulovesicular autophagosomes (TVAs)^37^. They pointed that polyplex-mediated gene transfection yield was reduced upon TVA generation, implying that autophagy can be a barrier for the gene delivery^37^.

Chaperone-mediated autophagy is a specific sort of autophagy which uses lysosomal receptors and chaperone proteins to immediately transfer target proteins inside the lysosomes, where active degradation occurs^47^. Target proteins display a penta-peptide motif (KFERQ) and are therefore selectively recognized by the cytosolic chaperone HSP70; that promotes target protein to be transferred to the lysosomes^48^. HSP70 inhibitors also inhibit autophagy^49^. Thus we used Pifithrin-μ, an HSP70 inhibitor, which significantly increased the splice correction activity and we also analyzed the possible interaction between PF14 and the Hsc70 using docking simulation approach (Fig. 7).

In wild-type macrophages, FAK is localized to the exterior of the Salmonella-containing vacuole (SCV), inducing signaling throughout the Akt-mTOR axis and hindering the autophagy induction. In FAK-deficient macrophages, Akt/mTOR signaling is suppressed, and the autophagic acquisition of intracellular bacteria is intensified, leading to a greater extent of bacteria degradation^50^. In our study, FAK inhibition dramatically reduced the splice correction activity, which is concordant with the higher autophagic degradation or sequestration of the SCO, similar to the case of Salmonella in FAK-deficient macrophages^50^.

Concerning mTOR pathway, to suppress the autophagy we activated mTOR, recording a significant increase in splice correction activity, in agreement with the fundamental role of mTOR in autophagy inhibition. Additionally, several nanoparticles have also been shown to downregulate this pathway^12,51–58^. Moreover, connected to the adhesion-related autophagy induction, inhibition of CTGF significantly decreased the splice correction by PF14 transfection system, in agreement with the reported effect of the loss of CTGF implying defective induction of the autophagic response, mediated by nuclear factor κB (NFκB) as part of a CTGF/integrin/NFκB signaling pathway^59^.

The β2-adrenergic receptor is a major activator of autophagy^60^, and we found that β2 antagonist led to a significant increase in the splice correction activity. Furthermore, autophagy induction by atorvastatin^61^ significantly reduced the splice correction activity mediated by PF14 transfection system. Prostaglandin E2 (PGE2) induces autophagy^62^ and is capable of diminishing splice correction as well.

Autophagy also has a principal function in controlling the metabolism of lipids, including cholesterol and stearic acid, this last one included in the PF14 structure. AHR ligands have also been reported to regulate lipid metabolism and cholesterol biosynthesis. In human macrophages, AHR activation has been demonstrated to induce lipid accumulation^63^. CH223191, an AHR antagonist, decreases the cell surface levels of tissue factors (TF), leading to sterol synthesis inhibition. This pathway could influence the autophagy mechanism^64^. Also, CH223191 increased the PF14 transfection system activity.

A significant part of delivery vehicles for gene therapy, including PF14 transfection system, aimed at targeting functional nucleic acids to the cell nucleus. The nuclear pore complex (NPCs) participates in nuclear autophagy, a standard response stimulated by DNA damage, and seems to be an essential route for clearing nuclei from damaged elements or extra chromosomes emerging from inaccurate mitoses. Mutants incomplete in nuclear autophagy display gene amplification, developed DNA damage, aneuploidy and chromosome imbalance. The nuclear envelope (NE) ruptures owing to an autophagy-like pathway in conventional tissue culture cells involve transient NE breaks that are promptly repaired^65^. A broad variety of proteins is transferred in both regions within the cytoplasm/ER and the nucleus while these breaks heal. These ruptures occur at prominent incidence during the virus infection^66–68^, which is rather a similar condition to the treatment with PF14 transfection. Moreover, inhibition of importin-β transport receptors enables higher splice correction activity through PF14 transfection system.

The application of gene therapy for clinical purposes, including gene delivery to manage genetic mutations, has been hindered by difficulties of delivery. The shortage of data about the genetic expression profiles and intracellular signalling pathways that are controlling transfection efficiency also pose limits to the design of optimal delivery vectors. This investigation is the first to demonstrate the autophagy induction and cellular effects triggered by cell penetrating peptide transfection system in basal cell signalling, with the aim to unravel the molecular basis for potent nucleic acid delivery and to use that knowledge to design better CPPs. PF14 transfection system is showing the high capacity to deliver nucleic acid combined with non-toxic autophagy induction, as a combinatory therapy strategy. For example, correction of mRNA splicing is critical to cure several types of muscular dystrophies and also to regulate proper autophagy induction. In the same way, PF14 transfection system in combination with autophagy inhibitors is a promising non-invasive strategy. Moreover, controlled autophagy induction is part of a vital innate immune defence system that can be manipulated as an anti-pathogenic defence system. In the case of PF14 transfection system, its ability to induce autophagy can be seen as an additive effect resulting in its ability to deliver the anti-pathogenic drug in sort of small molecules, proteins or nucleic acids. We showed that the splice correction activity could be significantly upregulated by transfecting the cells in combination with small ligand molecules able to suppress the autophagy process. Additional studies are required to understand and estimate the influences of different delivery systems on the autophagy system, as well as molecular interruptions on gene expression profiles of transfected cells to uncover and validate the pathways controlling the transfection process. However, a deeper understanding of gene expression profiles induced by different transfection systems, and better optimized non-viral delivery systems based on modulation of the activity of the signalling pathways are required in order to be able to designed and improve the therapeutic applicability.

## Electronic supplementary material

Supplementary Figure 1

Supplementary Table 1

Supplementary Table 2
