# Supplementary material for: Role of autophagy in cell-penetrating peptide transfection model
Source: Sci Rep. 2017 Oct 3;7:12635. doi: 10.1038/s41598-017-12747-z (PMC5626743; doi:10.1038/s41598-017-12747-z)
Supplement: Supplementary file 1 — Supplementary Figure 1 [file 41598_2017_12747_MOESM1_ESM.pdf]

Role of autophagy in cell-penetrating peptide transfection model

Moataz Dowaidar[a*], Maxime Gestin[a], Carmine Pasquale Cerrato[a], Mohammed Hakim Jafferali[a], Helerin Margus[b,c], Paula Ann Kivistik[d], Kariem Ezzat[e], Einar Hallberg[a], Margus Pooga[b], Mattias Hällbrink[a], and Ülo Langel[a,c*]

[a]Department of Neurochemistry, The Arrhenius Laboratories for Natural Sciences, Stockholm University, Svante Arrhenius väg 16B, SE-10691 Stockholm, Sweden.

[b]Department of Developmental Biology, Institute of Molecular and Cell Biology, University of Tartu, 23 Riia Street, 51010, Tartu, Estonia

[c]Laboratory of Molecular Biotechnology, Institute of Technology, University of Tartu, Nooruse, 50411 Tartu, Estonia.

[d]Estonian Genome Center, University of Tartu, Tartu, Estonia.

[e]Department of Laboratory Medicine, Karolinska Institutet, Huddinge, Sweden.

*Corresponding authors at Dpt. Neurochemistry, Stockholm University, S.Arrheniusv. 16B, SE-106 91 Stockholm, Sweden. Moataz Dowaidar, E-mail address: moataz@neurochem.su.se, Ülo Langel, Tel: +46-8-161 793; Fax: +46-8-161 371; E-mail: ulo@neurochem.su.se.

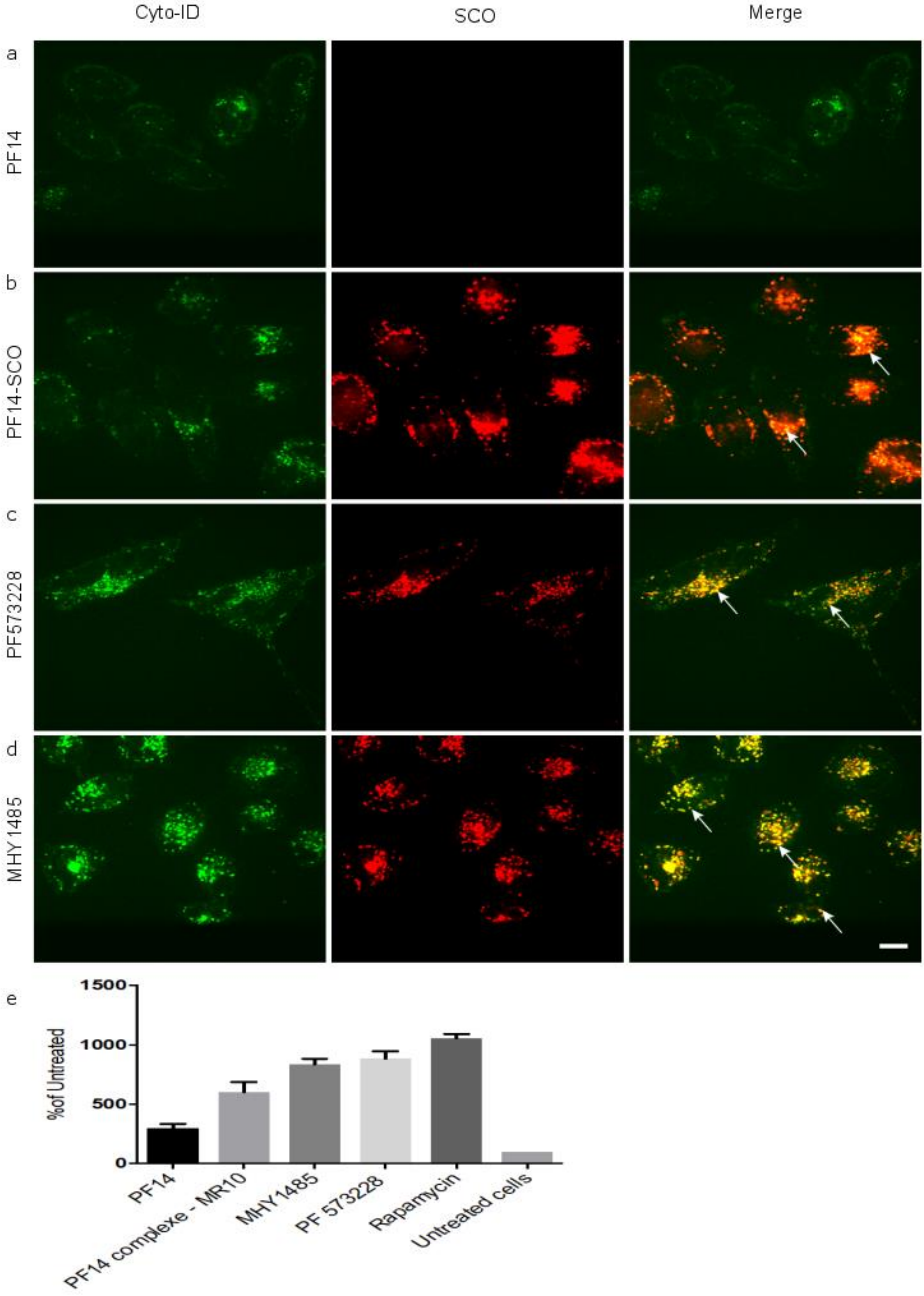

Cyto-ID
SCO
Merge
a
PF14
b
PF14-SCO
c
PF573228
d
MHY1485
e
1500
1000
500
0
%of Untreated
PF14
PF14 complexe - MR10
MHY1485
PF 573228
Rapamycin
Untreated cells

Supplementary Figure 1: Induction of autophagy by PF14 and its complexes with SCO in HeLa cells using detection by Cyto-ID staning. Confocal micrographs of HeLa-pLuc705 cells treated with 2 µM PF14 (a), PF14-SCO-Alexa 568 nano-complexes (red, formed at molar ratio 10/1) for 24 h (b), and autophagosomes stained with Cyto-ID (green). The cells in c and d were treated before addition of PF14-SCO complexes with (c) 6 µM FAK inhibitor (PF573228) or (d) 10 nM mTOR activator (MHY1485) for 1 h. The fluorescence signal intensity levels were adjusted for better visibility. (e) Quantification of Cyto-ID signal from HeLa-705 cells in response to treatment with PF14, its complexes with SCO at MR 10, PF14 complexes with 10 nM mTOR activator (MHY1485) or 6 µM focal adhesion kinase (PF573228) inhibitor, Scale bar is 10 µm.
